# Natural Killer Cells Infiltration in the Joints Exacerbates Collagen-Induced Arthritis

**DOI:** 10.3389/fimmu.2022.860761

**Published:** 2022-03-30

**Authors:** Lisheng Wu, Ran Wang, Yi Zhou, Di Zhao, Feilong Chen, Xianghui Wu, Xiaoguang Chen, Shixian Chen, Juan Li, Junqing Zhu

**Affiliations:** ^1^ Department of Rheumatic & TCM Medical Center, Nanfang Hospital, Southern Medical University, Guangzhou, China; ^2^ Department of Traditional Chinese Internal Medicine, School of Traditional Chinese Medicine, Southern Medical University, Guangzhou, China; ^3^ Department of Obstetrics, Guangdong Women and Children Hospital, Guangzhou, China; ^4^ Laboratory Animal Research Center, Nanfang Hospital, Southern Medical University, Guangzhou, China; ^5^ Department of Pathogen Biology, Key Laboratory of Tropical Disease Research of Guangdong Province, School of Public Health, Southern Medical University, Guangzhou, China

**Keywords:** rheumatoid arthritis, collagen-induced arthritis, natural killer cell, NKp46, adoptive cell transfer

## Abstract

**Introduction:**

The role of natural killer (NK) cells in rheumatoid arthritis remains controversial. We aimed to assess the role of NK cells in the pathogenesis of rheumatoid arthritis.

**Materials and Methods:**

The percentage of NK cells in the peripheral blood, spleen, lymph nodes and inflamed paws from collagen-induced arthritis mice were examined through the disease progression. Correlation between the proportion of NK cells and subsets with arthritis score, histopathological changes, and bone destruction were evaluated. Adoptive cell transfer was performed to determine the effect of NKp46^+^NK cells on arthritis development, and the role of receptor NKp46 was explored with NKp46 knockout mice.

**Results:**

The percentage of NK cells in peripheral blood decreased at the late stage of the disease and negatively correlated with arthritis score. NK cells increased in the inflamed paws during arthritis development and were positively associated with arthritis score, histopathological change, and bone destruction. Adoptive transfer of NKp46^+^NK cells before disease onset resulted in increased NK cells infiltration in the joints, higher incidence of arthritis, more severe clinical symptoms, and more pronounced joint inflammation and bone damage. NKp46 deficiency had no significant influence on the incidence and severity of arthritis in collagen-induced arthritis mice.

**Conclusions:**

NK cell infiltration in the joints positively correlates with arthritis progression, inflammation, and bone destruction. The pathogenic role of NK cells in rheumatoid arthritis may be independent of the receptor NKp46.

## Introduction

Rheumatoid arthritis (RA) is a chronic, systemic, inflammatory autoimmune disease characterized by joint inflammation, chronic synovitis, cartilage damage, and bone erosion (1). Though the etiology of RA is unknown, it is generally accepted that in susceptible individuals, post−translational modifications can trigger overt autoimmunity, followed by synoviocytes expansion and cytokines and proteases production, and infiltration of immune cells, e.g., neutrophils, monocytes/macrophages, and natural killer (NK) cells into the synovium (2).

NK cells, a subset of innate lymphoid cells (ILCs), possessing both cytotoxicity and cytokine‐producing properties, participate in many immune activities, e.g., control of viral infections, antitumor immunity, and autoimmune diseases (3–6). In humans, NK cells are generally divided into two major subsets, CD56^dim^CD16^+^ and CD56^bright^CD16^−/dim^ (7, 8). In mice, NK cells are defined as NK1.1^+^ cells, or DX5^+^ for circulating NK cells (CD49b, Integrin VLA-2α) in mouse strains that do not express NK1.1 (9, 10). In 2013, NK cells were classified as a group 1 innate lymphoid cell (ILC), comprising conventional NK cells and ILC1s (11). More recently, based on development and function, ILCs were classified into five subsets—NK cells, ILC1s, ILC2s, ILC3s, and LTi cells (3). According to this definition, NK cells are cytotoxic cells circulating in the bloodstream, whereas ILC1s are generally noncytotoxic or weakly cytotoxic with low perforin levels but produce high levels of cytokines like interferon (IFN)-γ, tumor necrosis factor (TNF)-α, and granulocyte–macrophage colony-stimulating factor (GM-CSF) (3, 11). ILC1s have some common phenotypic markers with NK cells, such as NKp46 in humans and mice. In this paper, for lucidity and convenience, we use the conventional term “NK cells” to refer to such ILCs.

The role of NK cells in RA has been evaluated in patients and animal models but with conflicting results. An association of expansion of NK cells with clinical response to Etanercept or Rituximab in RA patients was noted (12, 13). It was shown that noncytotoxic, cytokine-producing NK cells are expanded in the inflamed joints of RA and may serve as a marker for RA disease severity (14–18). However, reports on the effect of NK cells in animal models of RA are conflicting. Soderstrom et al. reported depletion of NK cells from collagen-induced arthritis (CIA) mice almost wholly prevented bone erosions. In contrast, Lo et al. documented an early onset of arthritis with more severe clinical symptoms upon NK cell depletion (19, 20). Recently, Louis et al. reported that synovial joint NK cells produce GM-CSF in an IL-18-dependent manner and propagate joint inflammation (15).

NKp46, also known as natural cytotoxicity receptor 1 (NCR1), a glycoprotein belonging to the immunoglobulin (Ig) superfamily, is expressed by NK cells, ILC1, one subset of ILC3 and a subset of γδ intraepithelial lymphocytes (3, 21). As one of the major activating receptors in humans and the only NCR in mice (22–24), NKp46 has a crucial role in the antitumor and antiviral activities and autoimmune settings of NK cells (25, 26). NK cell functions are regulated by a repertoire of receptors, namely, activating receptors NKG2D, CD16 (Fcγ receptor III), NKp30, NKp44, and NKp46; killer cell immunoglobulin‐like receptor; and the inhibitory receptors CD94/NKG2A and LY49 (mouse) (27). NKp46 mediates NK cell function in RA pathogenesis with other NK cell activating and inhibitory receptors. Researchers found that fibroblast-like synoviocytes in RA (RA-FLS) expressed ligands for NK cell receptors and stimulated degranulation of the human NK cell line Nishi cells towards RA-FLS, in which NKG2D, DNAM-1, NKp46, and NKp44 were the key activating receptors involved (28). On the other hand, activating NK cells through blockade of its inhibitory CD94/NKG2A receptor enhanced the elimination of pathogenic follicular T_H_ and T_H_17 cells and elicited arrest of CIA progression (29). Interestingly, Matos et al. reported masking of CD94/NKG2A upregulated IFN-γ and tumor necrosis factor production in activated synovial NK cells (30). While blockade of the activating receptor NKG2D ameliorated established CIA, which also reduced interleukin-17 production from CD4^+^ T cells and splenic NK cell cytotoxic effector functions (31). Signals delivered by the co-engagement of NKp46 with 2B4 strongly cooperate to enhance degranulation in total resting peripheral blood NK cells (32). Human NK cells can induce neutrophil apoptosis *via* an NKp46- and Fas-dependent mechanism (33). It is notable that though the role of NKp46 has been investigated intensively in innate immunity, the contribution of NKp46 to RA pathology remains largely unknown.

This study investigates the alteration of NK cells in CIA mice over disease development in different tissues and possible association with the severity of arthritis. We further examined the role of NK cells *via* cell transfer and the receptor NKp46 *via* NKp46 knockout mice.

## Materials and Methods

### Mice

DBA/1 and C57BL/6 mice were purchased from Pengyue Laboratory Animal Company (Jinan, China) and were housed in a specific-pathogen-free environment. NKp46 knockout C57BL/6 mice (*Ncr1* ^gfp/gfp^) were previously described (34) and were purchased from Jackson Laboratory (#022739). All mice used in these studies were 10–12 weeks of age. All procedures involving animals were performed following relevant ethical guidelines and were reviewed and approved by the Animal Care and Use Committee of Southern Medical University.

### Reagents

Bovine type II collagen, Chick type II collagen, Complete Freund’s Adjuvant, and Incomplete Freund’s Adjuvant were purchased from Chondrex (Redmond, WA). Purified anti-mouse CD16/32 antibody (101302), anti-CD49b (DX5, 108901) and fluorochrome-conjugated antibodies, anti-CD3ϵ-PerCP/Cy5.5 (145-2C11, 100328) and anti-CD49b-FITC (DX5, 108906) were purchased from Biolegend (San Diego, CA), while anti-CD335-BV421 (29A1.4, 562850) was purchased from BD Biosciences (San Jose, CA). Anti-CD335 antibody (PA5-102860), Alexa Fluor 488 goat anti-rat IgM (A-21212), and Alexa Fluor 594 goat anti-rabbit IgG (A-11007) were purchased from Invitrogen (Waltham, MA). Collagenase/Dispase (11097113001) was purchased from MilliporeSigma (Darmstadt, Germany). Vectashield antifade mounting medium with DAPI (H-2000) was purchased from Vector Lab (H-2000, Burlingame, CA).

### Induction of Collagen-Induced Arthritis

**Figure 1:**
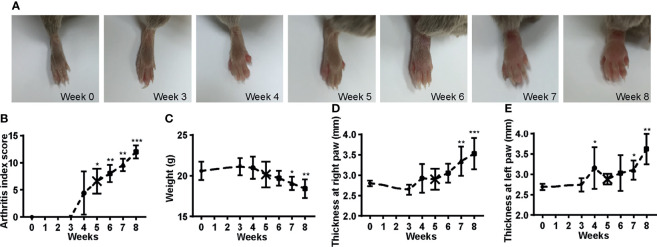
Development of CIA mice. **(A)** The hind paws show from mild to pronounced edematous swelling and eventually ankylosis over the disease course. **(B)** The arthritis score increased with time. **(C)** The body weight decreased over the onset of the disease. **(D–E)** The thickness of the hind paws increased over time. ANOVA with *post-hoc* Dunnett’s T3 test for **(B)** and ANOVA with *post-hoc* LSD test for **(C–E)**; *p < 0.05, **p < 0.01, ***p < 0.001 vs. week 0. n = 5 per group. Results are representative of three independent experiments.

CIA models were established in male DBA/1 mice (10 weeks old) by intradermally immunizing with 100 µl emulsion of Bovine type II collagen (2 mg/ml in 10 mM acetic acid) emulsified with an equal volume of Complete Freund’s Adjuvant (containing 4 mg/ml heat-inactivated *Mycobacterium tuberculosis*) at the base of the tail over two injection sites on day 0. A booster immunization was given on day 21 as on day 0 but with Bovine type II collagen emulsified with Incomplete Freund’s Adjuvant. Arthritis was monitored using the arthritis index scoring system as described (35): 0, normal; 1, swelling of 1 joint (wrist/ankle or digit); 2, swelling of 2 joints or more; 3, swelling of all joints; 4, swelling of all joints and ankylosis. The score for each paw was determined, resulting in a total score of 0–16 per mouse. Paw thickness was measured using a caliper, and body weight was recorded. The CIA mice were sacrificed on weeks 0, 3, 4, 5, 6, 7, and 8 after the first immunization separately for indicated analysis. The CIA mice developed arthritis 3 weeks after the initial vaccination and progressed over time, with paws showing mild to pronounced edematous swelling and eventually ankylosis (**Figure 1A**). Throughout the disease, the arthritis index score in these mice increased gradually and peaked on week 8 (**Figure 1B**), with bodyweight decreased, and the thickness of both hind paws increased and reached maximum extent on week 8 (**Figures 1C–E**). These indicated that the CIA model is working as expected. The same procedure was followed to induce CIA in *Ncr1* ^gfp/gfp^ and wild-type C57BL/6 mice, except that Chick type II collagen was used instead of Bovine collagen in both immunizations. Arthritis in *Ncr1* ^gfp/gfp^ and C57BL/6 mice was scored as (35): 0, normal; 1, mild swelling; 2, moderate swelling (or mild swelling + 1 or 2 swollen joints; 3, swelling of all joints; 4, joint distortion and/or rigidity and dysfunction.

### Flow Cytometric Analysis

The mice were euthanized on days 0 or 3, 4, 5, 7, or 8 weeks post first immunization, and single-cell suspension was prepared from peripheral blood, spleens, lymph nodes, and paws. Single-cell suspensions from paws were prepared according to Leavenworth et al. (29). Briefly, paws were dissected with fur striped, digested with collagenase/dispase for 1 h at 37°C, followed by filtration through 70 μm nylon meshes. Fc receptors were blocked with anti-CD16/CD32 antibodies, followed by staining with specific antibodies. Flow cytometry was performed with a FACS Aria II flow cytometer (BD Biosciences, San Diego, CA, USA) and analyzed using the FlowJo software (TreeStar, Ashland, OR, USA).

### Micro-Computed Tomography (Micro-CT) Assessment

When the mice were euthanized, the right hind paws from each mouse were removed and subjected to micro-CT analysis. Five mice from each time point underwent micro-CT scanning, which was then subjected to immunofluorescence and histological study. Samples were scanned with µCT 80 (Scanco Medical AG, Brüttisellen, Switzerland), utilizing beam parameters of 55 kVP and 145 µA, with an isotropic voxel size of 15 µm and integration time of 200 ms. Three-dimensional (3D) images were constructed, and bone mineral density (BMD) and bone volume/total volume (BV/TV) of the talus was calculated.

### Immunofluorescence and Histopathological Assessment

For immunofluorescence analysis, dissected hind paws were fixed with 4% paraformaldehyde and decalcified, then paraffin-embedded and sectioned at 4 μm thickness. Samples were blocked with 10% goat serum at room temperature followed by incubating overnight at 4°C with primary antibodies against NKp46 (1:100) and DX5 (1:100). The sections were incubated with fluorescent label conjugated secondary antibodies and counterstained with DAPI. Images were acquired with an Olympus BX51 microscope. The number of NKp46 and DX5 double fluorescent cells was determined by three random fields per section, three sections per mouse, and five mice per time point. For histopathological assessment, samples were stained with hematoxylin–eosin (H&E), and histological changes were evaluated blindly by two researchers using three parameters: inflammation was scored based on the infiltration of inflammatory cells; synovial hyperplasia was scored based on the increase of the cellularity of the synovial membrane and synovial thickening; bone and cartilage destruction was scored based on the loss of articular cartilage and the erosion of cortical bone. Each parameter ranged from 0 to 3, with 0 representing no change and higher scores indicating a more severe change. The individual parameters were summed up to calculate the total score.

### Adoptive Cell Transfer

FACS sorted NKp46^+^NK cells from peripheral blood of CIA mice 5 weeks post first immunization was transferred to CIA mice through the tail vein during the second immunization on week 3. These CIA mice were randomized into 4 groups: receiving 1 × 10^5^ (Low-week 5) or 1 × 10^6^ (High-week 5) NKp46^+^NK cells and euthanized on week 5 post first immunization; receiving PBS and euthanized on week 5 (PBS-week 5) or 8 (PBS-week 8) post first immunization.

### Statistical Analysis

All statistical analyses were conducted using SPSS version 25.0 (SPSS Inc., Chicago, IL, USA) and GraphPad Prism version 8 (GraphPad Software, San Diego, CA, USA). Continuous data were presented as mean ± S.D., and frequency data as numbers (n) and percentages (%). For continuous data, Shapiro–Wilk and Levene’s test were applied to evaluate normality and homogeneity of variance, respectively. For normally-distributed variables, Student’s t test or one-way analysis of variance (ANOVA was used to assess the differences as appropriate. Depending on the homogeneity of variances, either Dunnett’s T3 test or Fisher’s least significant difference (LSD) test was chosen for *post hoc* analysis. For non-normally-distributed continuous data, Mann–Whitney U test or Kruskall–Wallis test was applied. Correlations were determined by Pearson correlation analyses should the variables be normally distributed, or otherwise by Spearman analyses. The log-rank test was used to compare incidence rates of arthritis between *Ncr1* ^gfp/gfp^ and wild-type C57BL/6 mice, and two-way repeated measures ANOVA for arthritis index score (Greenhouse–Geisser adjustment was used when sphericity violated.). A *p*-value <0.05 was regarded as statistically significant.

## Results

### NK Cells Infiltration in Inflamed Paws Positively Correlates With Arthritis Progression

**Figure 2:**
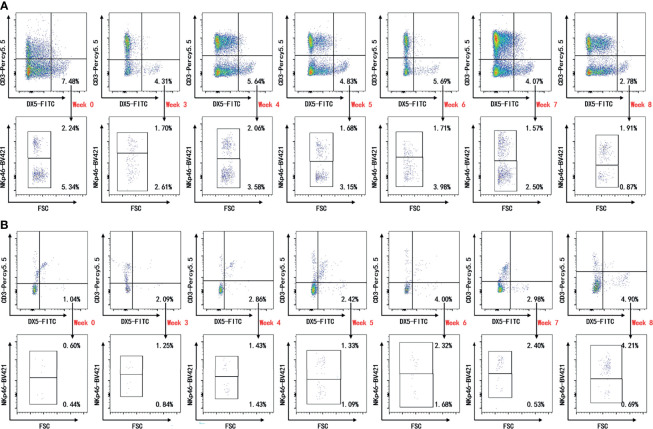
Representative plots of cytometric analysis. Flow cytometric analysis for indicated time points. Single live lymphocytes in peripheral blood **(A)** and paws **(B)** gated on CD3^−^DX5^+^ were further gated for NKp46. The gates were set upon isotype controls.

**Figure 3:**
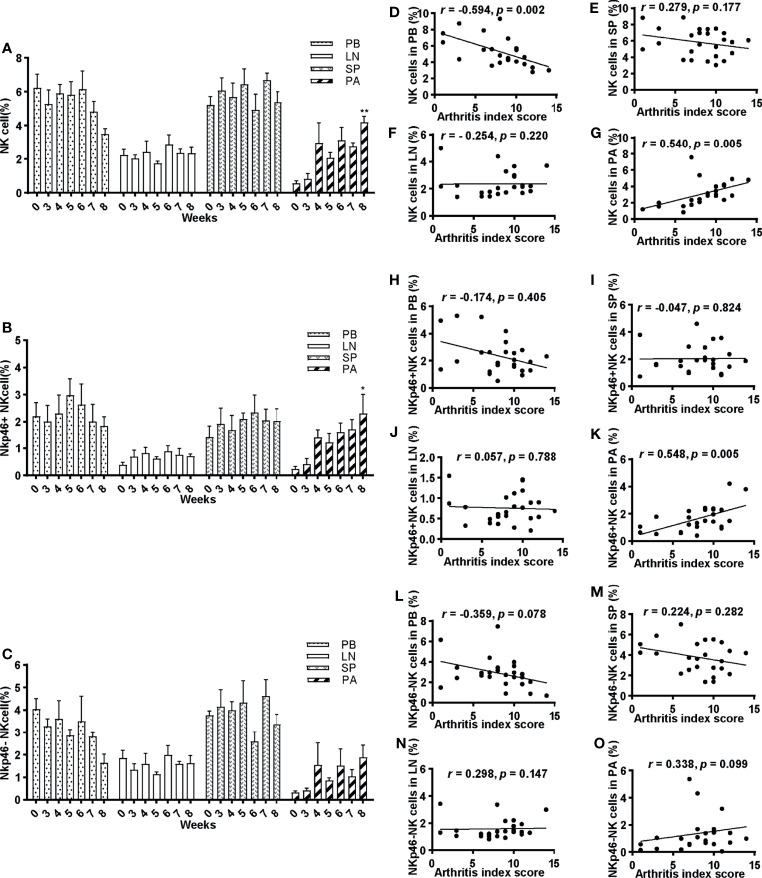
Frequencies of NK cells and their correlations with arthritis index score. **(A–C)** Percentage of NK cells, NKp46^+^NK cells, and NKp46^−^NK cells of lymphocytes in peripheral blood, spleen, lymph nodes, and paws at 0, 3, 4, 5, 6, 7, and 8 weeks post first immunization, respectively. ANOVA was used for comparing NK cells in PB and SP; NKp46^+^NK cells in LN and SP; NKp46^-^NK cells in PB; others by Kruskall–Wallis test. *p < 0.05, **p < 0.01 after adjusted by the Bonferroni correction vs. week 0. PB, peripheral blood; LN, lymph nodes; SP, spleen; PA, paws. **(D–O)** Correlations of NK cells, NKp46^+^NK cells, and NKp46^−^NK cells in peripheral blood, spleen, lymph nodes, and paws with arthritis index scores were explored. Pearson correlation analyses were used for D, F, G, I, L, N, and Spearman analyses for others. n = 5 per group. Results are representative of three independent experiments.

Peripheral blood, spleen, draining lymph nodes, and hind paws were collected on weeks 0, 3, 4, 5, 6, 7, and 8 after the first immunization for flow cytometric analysis. Representative plots are shown in **Figure 2** and **Supplementary Figure 1** (**Figure 2** and **Supplementary Figure 1**). Decreased levels of CD3^−^DX5^+^ cells and CD3^−^DX5^+^NKp46^−^ cells on week 8 were detected in the peripheral blood compared with week 0, though not statistically significant (**Figures 3A, C** **)**. In comparison, no significant changes in the frequencies of CD3^−^DX5^+^ cells and NKp46 subsets were observed at any time points analyzed in spleen and lymph nodes (**Figures 3A-C**). In the paws, the percentages of CD3^−^DX5^+^ cells and CD3^−^DX5^+^NKp46^+^ cells increased with disease progression and reached the greatest extent on week 8 (**Figures 3A–C**).

The correlation between NK cells and their subsets and arthritis index score was explored. There was a statistically significant moderate correlation (r = 0.540, p = 0.005) between frequencies of NK cells and arthritis index score in the paws, and a reverse correlation in the peripheral blood (r = −0.594, p = 0.002) (**Figures 3G, D** **)**. In contrast, no significant correlations in the spleen and lymph nodes were noted (**Figures 3E, F** **)**. Similarly, a positive correlation was found between NKp46^+^NK cells and arthritis index score in the paws (r = 0.548, p = 0.005) (**Figure 3K**), whereas no correlations in peripheral blood and lymphoid tissues (**Figures 3H–J**). As for NKp46^−^NK cells, no statistically significant correlations were identified in any tissue explored (**Figures 3L–O**).

### NK Cells Infiltration in Inflamed Joints Positively Correlates With Inflammation and Bone Erosion During the Progression of CIA

**Figure 4:**
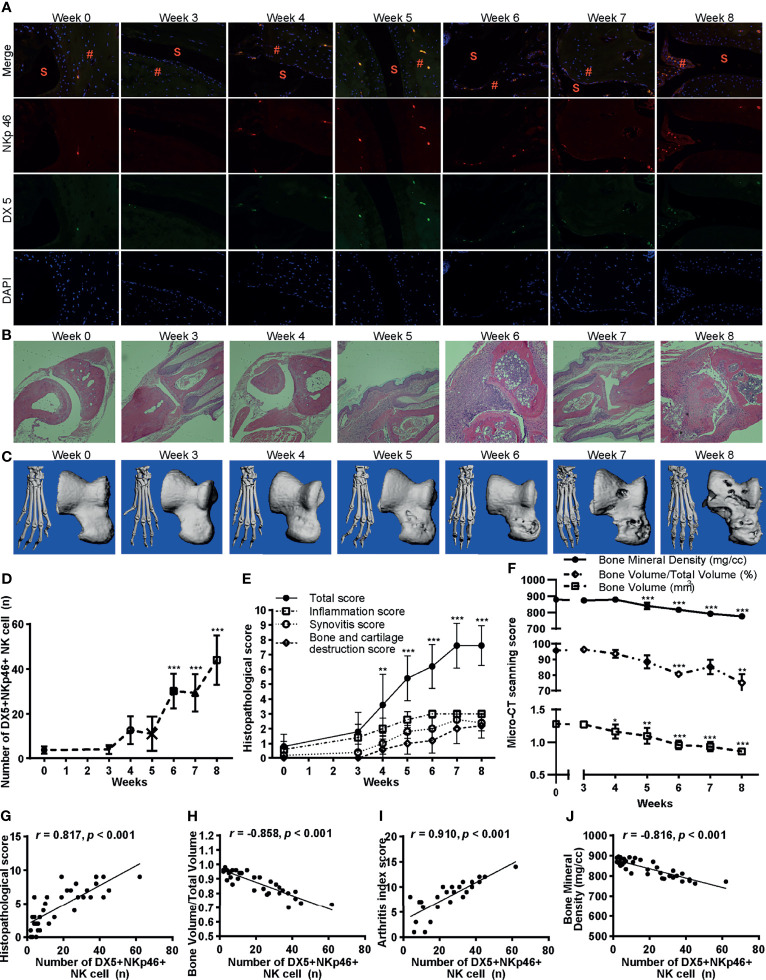
Infiltration of NKp46^+^NK cells correlates with inflammation and bone erosion. **(A)** Representative images of immunofluorescence examination of NKp46^+^NK cells in hind paws. Cells were stained with anti-NKp46 (red) and anti-DX5 antibodies (green), and nuclei were stained with DAPI (blue), ×100 magnification. S, synovial space; #, synovial tissue. **(B)** Representative histological staining (H&E, ×100 magnification). **(C)** Reconstructed micro-CT 3D models of the claw (left) and talus (right). **(D–F)** Changes of the number of DX5^+^NKp46^+^NK cells, histopathological score, and micro-CT quantitative measurements of bone destruction over disease progression, respectively. ANOVA with *post-hoc* LSD test for all except that ANOVA with *post-hoc* Dunnett’s T3 test for bone volume/total volume. *p < 0.05, **p < 0.01, ***p < 0.001 vs. week 0. n = 5 per group. **(G–J)** Correlations of the DX5^+^NKp46^+^NK cells in the joints with histopathological score, bone volume to total volume, arthritis index score, and bone mineral density, respectively. Spearman correlation analyses were used. n = 5 per group. Results are representative of three independent experiments.

We further examined the infiltration of NK cells in the joints by immunofluorescence staining of NKp46 and DX5 double fluorescent cells (**Figure 4A**). Consistent with the cytometric findings, the number of NKp46^+^NK cells in the joints increased with the development of the disease and reached maximum extent on week 8 (**Figure 4D**). The histological analysis investigated by H&E stains of the hind paws showed that the severity of inflammation, synovitis, bone erosion, and cartilage damage also exacerbated over time (**Figures 4B, E** **)**. Bone destruction was evaluated using micro-CT. The claw, namely, the ankle joints and talus, was reconstructed, and bone destruction was observed over the onset of the disease (after week 3) (**Figure 4C**). The bone surface became cruder, and bone volume loss more evident as the disease progressed (**Figure 4C**). Quantitative measurements of the talus bone destruction showed that bone mineral density and bone volume to total volume significantly decreased over disease progression (**Figure 4F**).

Next, we inspected the correlation of infiltration of NKp46^+^NK cells in the joints with histological changes, arthritis index score, and bone destruction. As expected, the number of NKp46^+^NK cells in the joints highly correlated with the histopathological score, bone volume to total volume, arthritis index score, and bone mineral density (r = 0.817, −0.858, 0.910, and −0.816 respectively; with each p <0.001) (**Figures 4G–J**).

### Adoptive Transfer of NK Cells Exacerbate Arthritis

**Figure 5:**
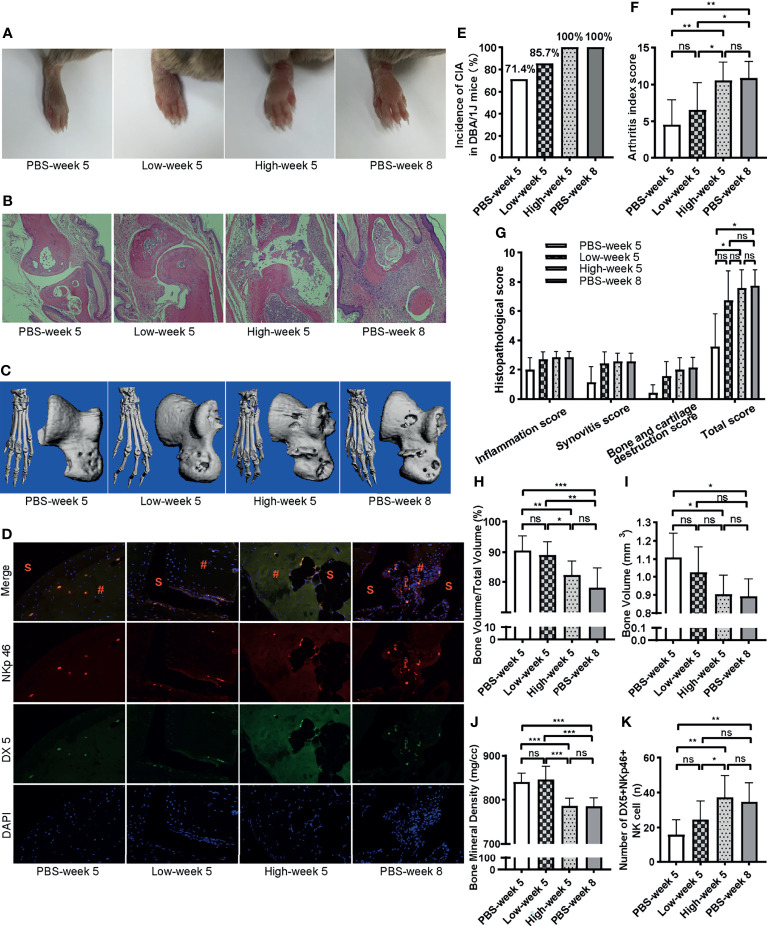
Adoptive transfer of NKp46^+^NK cells exacerbates arthritis. **(A)** Representative images of hind paws showing mild, moderate, and severe, severe swelling (from left to right). **(B)** Representative histological staining (H&E, ×100 magnification). **(C)** Reconstructed micro-CT 3D models of claw and talus. **(D)** Representative images of immunofluorescence examination of NKp46^+^NK cells in hind joints. S, synovial space; #, synovial tissue. **(E–K)** Incidence of arthritis, arthritis index score, histopathological score, bone volume, bone volume to total volume, bone mineral density, number of DX5^+^NKp46^+^ cells among different groups, respectively. ANOVA with *post-hoc* LSD test for **(F, H, J, K)**, *p < 0.05, **p < 0.01, ***p < 0.001, ns, not significant. Kruskall–Wallis test for **(G, I)**, *p < 0.05, ns, not significant after adjusted by the Bonferroni correction. n = 7 per group. Results are representative of three independent experiments.

To evaluate the pathogenicity of NKp46^+^NK cells in the progress of CIA, we performed adoptive cell transfer of NKp46^+^NK cells sorted by FACS (purity >90%) from peripheral blood of CIA mice 5 weeks after the initial immunization. Sorted NKp46^+^NK cells were transferred to CIA mice on week 3 during the second immunization. On week 5, both mice received 1 × 10^5^ (group Low-week 5) and 1 × 10^6^ (group High-week 5) NKp46^+^NK cells developed more severe arthritis compared with mice received PBS (group PBS-week 5). Mice from group High-week 5 had more severe arthritis than group Low-week 5 and were comparable to mice that received PBS and developed CIA till week 8 (group PBS-week 8) (**Figure 5A**). The incidence of arthritis was highest in the High-week 5 group, which reached 100% on week 5, with the Low-week 5 group more elevated than the week 5 control group (**Figure 5E**). The same trend was observed with arthritis index scores among these groups, though there was no statistically significant difference between Low-week 5 and PBS-week 5 groups (**Figure 5F**). In line with the arthritis index score, both the High and Low-week 5 and PBS-week 8 group showed prominent infiltration of inflammatory cells, synovial hyperplasia, and bone and cartilage destruction, with the moderate and mildest extent in the Low-week 5 and PBS-week 5 group, and most prominent in the other two groups, respectively (**Figure 5B**). The quantification of histopathological changes confirmed these observations. The total histopathological score in the PBS-week 5 group was the lowest, while no significant differences were observed among the other three groups (**Figure 5G**). Next, we examined the bone destruction in the claw and talus (**Figure 5C**). Compared to PBS-week 5 group, bone volume, bone volume to total volume, and bone mineral density decreased most significantly in the High-week 5 group, which had similar destruction with PBS-week 8 group. Yet, no differences were observed between group Low-week 5 and PBS-week 5 (**Figures 5H–J**). Comparing the High and Low-week 5 group, the higher dosage group had lower bone volume to total volume and bone mineral density (**Figures 5H–J**). Lastly, we inspected the infiltration of DX5^+^NKp46^+^ cells in joints by immunofluorescence. As expected, more DX5^+^NKp46^+^ cells infiltrated in the NKp46^+^NK cells transferred mice than the week 5 control mice (**Figure 5D**). The High-week 5 group had a greater magnitude of DX5^+^NKp46^+^ cells infiltration than the Low-week 5 and control group, and both NKp46^+^NK cells transferred groups displayed comparable infiltration with group PBS-week 8 (**Figure 5K**).

### NKp46 Deficiency Had no Significant Influence on the Incidence and Severity of Arthritis in CIA Mice

**Figure 6:**
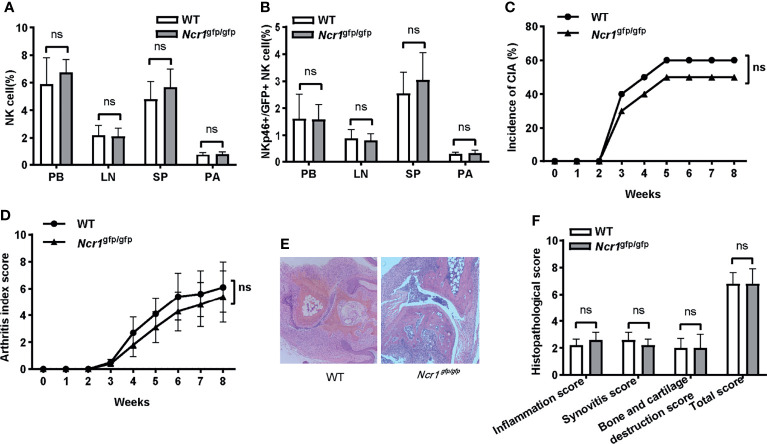
The impact of NKp46 deficiency on the incidence and severity of arthritis in CIA mice. **(A, B)** Frequencies of NK and NKp46^+^/GFP^+^NK cells in wild-type C57BL/6 and *Ncr1* ^gfp/gfp^ mice among different tissues. Student’s t-test; n = 5 per group. **(C)** Incidence of arthritis in wild-type C57BL/6 and *Ncr1* ^gfp/gfp^ mice. Log-rank test, n = 10 per group. **(D)** Arthritis index score in wild-type C57BL/6 and *Ncr1* ^gfp/gfp^ mice. Two-way repeated measures ANOVA. There is no significant group nor a time (week) × group effect. n = 10 per group. **(E, F)** Representative histological staining (H&E, ×100 magnification) and histopathological analysis between wild-type C57BL/6 and *Ncr1* ^gfp/gfp^ mice. Mann–Whitney U test for comparison of inflammation score, synovitis score, and total score; Student’s t-test for bone and cartilage destruction score; n = 5 per group. PB, peripheral blood; LN, lymph nodes; SP, spleen; PA, paws; ns, not significant. Results are representative of three independent experiments.

An important question is whether the function of NKp46^+^NK cells depends on the receptor NKp46. To answer this question, we assessed the incidence and severity of arthritis in an NKp46 knockout mouse model, *Ncr1* ^gfp/gfp^, in which NKp46 was replaced with a green fluorescent protein. First, we evaluated whether knocking out of NKp46 affected the development of NK and GFP^+^NK cells in *Ncr1* ^gfp/gfp^ mice. Both cell populations were assessed among different tissues, and no differences were found between wild-type C57BL/6 and *Ncr1* ^gfp/gfp^ mice (**Figures 6A, B** **)**. There was no significant difference in the onset and overall incidence of arthritis upon CIA induction (**Figure 6C**). Similarly, though the arthritis index score was lower in *Ncr1* ^gfp/gfp^ mice over the disease progress, the difference was not statistically significant (**Figure 6D**). No notable differences were found in the histopathological analysis between these two groups (**Figures 6E, F**).

## Discussion

NKp46 is found on resting and activated NK cells, and it was once accepted that NKp46 is expressed preceding the expression of DX5 at the immature NK cell stage (36). Later, researchers found that in the bone marrow, a major NK cell population expresses DX5 but not NKp46 and DX5^+^NKp46^−^NK cells are the precursors of DX5^+^NKp46^+^ cells (37). Here we define the NK cells in DBA1 mice as CD3^-^DX5^+^ cells.

The association of NK cells and RA disease activity has been investigated for decades. While some studies recognized increased CD56^+^CD3^−^ NK cells in peripheral blood in RA patients (17, 18), others described reduced NK cell count (12). Another study showed that circulating NK cells in RA patients were reduced and had compromised functions (38). This study showed a significant decrease of NK cells in peripheral blood at week 8, which represented the late stage of the disease, compared with week 0. Although not statistically significant, we observed a decrease–increase–decrease trend of NK cells in peripheral blood over the disease progression. This change may reflect the NK cell response upon disease stress and explain the difference of reported NK cell changes in RA patients, which may be affected by disease activity. The percentage of NK cells in the peripheral blood of deformative RA (DRA) patients was doubled compared to healthy controls and non-deformative RA (NDRA) patients (17). In recently diagnosed seropositive RA patients, the number of total NK cells was significantly decreased compared to healthy control. In contrast, seronegative RA patients showed an increase of CD56^bright^ NK cells in absolute numbers and frequencies (39). Schwaneck et al. documented rising numbers of NK (CD56^+^, CD3^−^, CD14^−^) cells with age in RA patients in the peripheral blood and those with current TNF-inhibition therapy (40), which demonstrated the complex factors influencing the status of NK cells. Interestingly, we observed a moderate negative correlation of NK cells in peripheral blood with arthritis index score, which agreed with Lin et al., who showed that RA disease activity (DAS28) exhibited an inverse correlation with the percentages of CD56^+^CD3^−^ NK cells (18). Chalan et al. also found that the proportion of CD56^+^ CD3^-^ NK cells were inversely correlated with DAS28 at baseline. The proportion of NK cells at baseline predicted DAS28 disease remission and was inversely correlated with the change in DAS28 after three months of tocilizumab (an IL-6 receptor inhibitor) treatment (41).

NKp46 is an activating receptor highly conserved in mammals and plays a role in non-major histocompatibility complex-restricted cytolysis mediated by NK cells (42). Besides its function in antitumor and antiviral activities, NKp46 plays a role in autoimmune settings (25, 26). It was found that NKp46 expression was reduced on peripheral blood NK cells in RA patients with bone deformity and erosion, and RA disease activity (DAS28) showed an inverse correlation with the percentages of CD56^+^CD3^−^ NK cells and NKp46 expression on NK cells (18). However, in this study, we did not find significant changes in NKp46^+^NK cells in the peripheral blood over the disease development nor correlated with arthritis index score. Though a decreased NKp46^-^NK cells, which may be precursors of NKp46^+^ cells (37), was found on week 8 in the peripheral blood compared to week 0, the correlation of NKp46^-^NK cells with arthritis index score was not observed. Moreover, no significant changes of NK, NKp46^+^NK, or NKp46^-^NK cells in the spleen or lymph nodes were found, which may reflect that the immunity abnormality of NK cells might be anatomical sites limited.

In line with investigations showing NK cells expanded in the inflamed joints of RA (14–18), we found an increase of total NK cells and NKp46^+^NK cells in paw tissues over the development of arthritis, both of which correlated positively with the arthritis index score. This is in line with the findings of Yamin et al., who showed that the percentage of NK cells within the synovial fluids has a trend of correlation with disease activity (DAS28-CRP) (17). We next confirmed the infiltration of NKp46^+^NK cells in joints by immunofluorescence, and following the cytometric analysis, a high positive correlation between NKp46^+^NK cells and arthritis index score was recognized. Furthermore, NKp46^+^NK cells were highly correlated with the histopathological score, which reflected joint inflammation, synovial hyperplasia, and bone and cartilage destruction. As a consequence of bone destruction, bone volume and bone mineral density decreased over disease progression and correlated negatively with the percentage of NKp46^+^NK cells. It was found that compared to those in the blood, NK cells from the synovial fluids had significant increases in NKp46 expression in both erosive deformative RA and non-deformative RA patients (17). Taken together, NK cells, especially NKp46^+^NK cells, have a pathogenic role in the development of RA.

The role of NK cells in the pathogenesis of inflammatory arthritis has been investigated intensively in past decades. In synovial fluid from OA patients, the presence of the CD56(+)^bright^CD16(−) cells expressing granzyme A correlated with increased levels of pro-inflammatory cytokines (43). In contrast, in systemic juvenile idiopathic arthritis, CD56(bright) NK cells have decreased granzyme K expression and IL-18-driven IFN-γ production (44), which demonstrated the multi-function role of NK cells in autoimmune arthritis. NK cells can secrete pro-inflammatory cytokines such as TNF-α and IFN-γ and can cause the activation or inhibition of other cells such as dendritic cells in a contact-dependent way (45, 46). CD3^-^CD56^bright^ NK cells from synovial fluid of RA patients can induce the differentiation of monocytes into potent T-helper-1 promoting dendritic cells (47). Furthermore, NK cells can engage with CD14^+^ monocytes in a reciprocal activatory manner and amplify the inflammatory response. When activated by IL-12, IL-15, and IL-18, CD56^bright^ NK cells promote TNF-α production by CD14^+^ monocytes, which conversely promote IFN-γ production by these NK cells (48). Moreover, NK cells can co-stimulate B and T cells and trigger cytokine production by fibroblast-like synoviocytes. The interaction of NK cells with FLS leads to the production of pro-inflammatory chemokines, cytokines, and MMPs (49). NK cells in the inflamed joints of patients with RA express both RANKL and M-CSF and can induce osteoclast differentiation of monocytes (19). It was shown that IFN-γ regulates susceptibility to CIA in mice through the suppression of IL‐17 (50, 51). It can induce rapid degradation of TRAF6, the RANK adapter protein, and therefore strongly suppresses osteoclastogenesis (52). However, reports on the expression of IFN-γ by NK cell is inconsistent. In recently diagnosed seropositive RA, NK cells from blood showed decreased IFN-γ expression (39), while NK cells from blood in deformative RA secrete more IFN-γ upon exposure to IL-2 compared with healthy blood, and synovial fluid NK cells from deformative RA secrete more IFN-γ stimulated with IL-2 or IL-15 than non-deformative RA (17). Lo et al. showed that NK cells from the spleen of CIA mice showed markedly reduced expression of IFN-γ, and normal NK cells strongly suppressed production of Th17 cells *via* IFN-γ production, suggesting that NK cells play a protective role in the development of CIA (20).

This study explored whether the infiltrated NKp46^+^NK cells had a pathogenic role in CIA with adoptive cell transfer. Studies on the effect of NK cell depletion in CIA are conflicting. Soderstrom et al. reported prevention of bone erosions, whereas Lo et al. documented exacerbated arthritis (19, 20). Our current work showed that the transfer of NKp46^+^NK cells caused a higher incidence of disease at an earlier time point, more severe arthritis index score and bone destruction in a cell-number dependent manner. Even low dosage of cells transferred caused almost the exact extent of histopathological changes on week 5 as those presented in the late stage of the disease without cell transfer. Indeed, Louis et al. demonstrated that, in serum transfer-induced arthritis (STIA) or CIA model, mature NK cells (CD127^–^ NKp46^+^ CD49b^+^) were recruited to inflamed joints and were activated by IL-1 family member IL-18 to produce GM-CSF and promoted autoantibody-induced inflammatory arthritis through GM-CSF (15). They also showed reduced disease severity at later time points in *Mcl1* ^fl/fl^:*Ncr1*-Cre and anti-NK1.1-treated WT mice (15). Consistent with their results, in this study, we showed direct evidence of the pathogenic role of NKp46^+^NK cells in CIA through cell transfer.

In light of the importance of NKp46^+^NK cells in CIA, we assessed the role of the receptor NKp46 in such pathogenesis. We found that deficiency of NKp46 had no substantial influence on the incidence and severity of arthritis, which indicated that the pathogenic function of NKp46^+^NK cells in CIA might not be dependent on or not be solely reliant on NKp46. Yet recent studies reported that NKp46-deficient mice lack constitutive expression of TNF-α-related apoptosis-inducing ligand (TRAIL) on ILC1s (53, 54). Since TRAIL has been implicated in the regulation of inflammation and bone destruction in RA (55–57), the current results of NKp46 knockout mice should be translated with prudence. Louis et al. demonstrated that synovial joint NK cells propagate joint inflammation by secreting GM‐CSF, which signals and recruits inflammatory cells into the joints (15). Their work elucidated a possible mechanism of how NK cells contribute to arthritis. Yet future studies to better understand the role of other cytokines and the NK cells activating receptors in RA are needed.

Though we showed adoptive transfer of NK cells exacerbated arthritis, whether NK cells infiltrating the joints and propagating the disease were recruited from circulation remains under further investigation. The limitation of this study includes not distinguishing between NK cells in the joints and the paw tissues by flow cytometry. Other methods to determine the number of NK cells in the joints besides the immunofluorescence experiment would better support our conclusions. Also, the absolute numbers of NK cells in other tissues were not assessed, which might likewise influence the development of CIA.

## Conclusions

This study examined the proportion of NK cells in the peripheral blood, spleen, lymph nodes, and paw tissues in CIA mice and their correlation with disease severity. We confirmed that infiltration of NK cells in CIA joints positively correlates with arthritis progression, inflammation, cartilage erosion, and bone destruction. Most importantly, we revealed the pathogenic role of NKp46^+^NK cells in rheumatoid arthritis through adoptive cell transfer, which prominently exacerbates CIA arthritis. NKp46 may not be the primary actor in the pathogenic function of NK cells in CIA. Overall, our current work suggests that NKp46^+^NK cells infiltrate in inflamed joints and participate in the pathogenesis of autoimmune arthritis.

## Data Availability Statement

The original contributions presented in the study are included in the article/**Supplementary Material**. Further inquiries can be directed to the corresponding authors.

## Ethics Statement

The animal study was reviewed and approved by the Animal Care and Use Committee of Nanfang Hospital, Southern Medical University.

## Author Contributions

JZ, JL, and SC contributed to conception and design of the study. LW, RW, and XW performed mouse experiments. LW, RW, and JZ performed flow cytometric analysis, immunofluorescence and histopathological assessment, and prepared the manuscript. DZ, YZ, and FC performed micro-CT analysis. XC provided critical advice on experimental conduction and revised the first draft of the manuscript. All authors listed have made a substantial, direct, and intellectual contribution to the work and approved it for publication.

## Funding

This study was supported by the Natural Science Foundation of China (Nos. 81803932 and 82174171) and the Natural Science Foundation of Guangdong Province (Nos. 2018030310025 and 2017A030313868). The funders had no role in study design, data collection, analysis, decision to publish, or manuscript preparation.

## Conflict of Interest

The authors declare that the research was conducted in the absence of any commercial or financial relationships that could be construed as a potential conflict of interest.

## Publisher’s Note

All claims expressed in this article are solely those of the authors and do not necessarily represent those of their affiliated organizations, or those of the publisher, the editors and the reviewers. Any product that may be evaluated in this article, or claim that may be made by its manufacturer, is not guaranteed or endorsed by the publisher.
