# Supplementary material for: Natural Killer Cells Infiltration in the Joints Exacerbates Collagen-Induced Arthritis
Source: Front Immunol. 2022 Mar 30;13:860761. doi: 10.3389/fimmu.2022.860761 (PMC9005809; doi:10.3389/fimmu.2022.860761)
Supplement: Supplementary file 1 [file Image_1.pdf]

Supplementary Material

1 Supplementary Figures

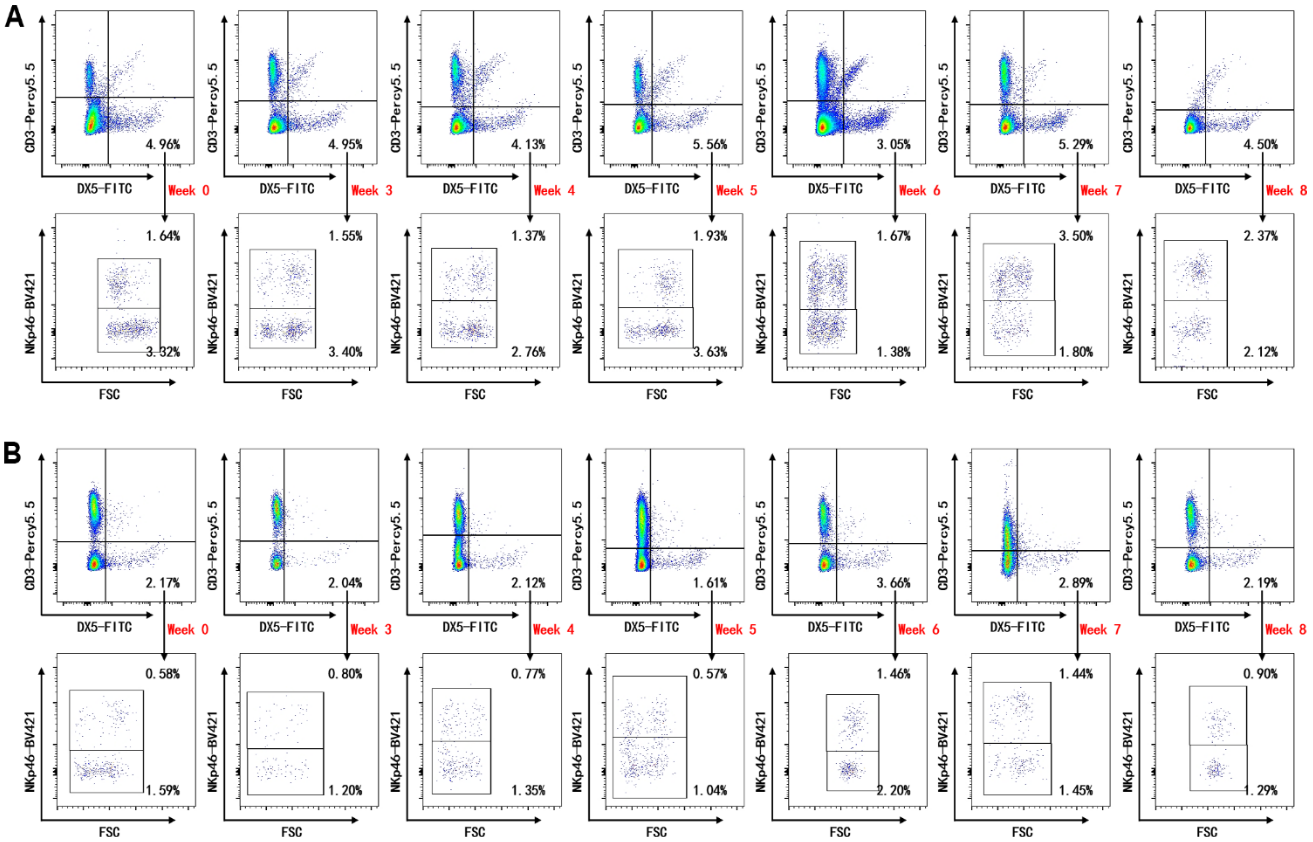

Supplementary Figure 1. Representative plots of cytometric analysis. Flow cytometric analysis for indicated time points. Single live lymphocytes in spleen **(A)** and lymph nodes **(B)** gated on $CD3^{-}DX5^{+}$ were further gated for NKp46. The gates were set upon isotype controls.
